# Multimodal In Vivo Imaging Reveals BZBS Capsules Enhance Brain Function and Delay Aging in the Mouse Models

**DOI:** 10.14336/AD.2025.0469

**Published:** 2026-06-06

**Authors:** Jiwei Xing, Xiufei Gu, Xin Chen, Guangxing Wang, Huiling Wu, Fengxian Du, Peishan Li, Heng Zhang, Ronghui Li, Yunlong Hou, Qingliang Zhao

**Affiliations:** ^1^The State Key Laboratory of Vaccines for Infectious Diseases, Xiang An Biomedicine Laboratory, Department of Medical Oncology, Department of ICU, Xiang’an Hospital of Xiamen University, School of Medicine, School of Public Health, Xiamen University, Xiamen, China.; ^2^Department of Orthopedics and Traumatology of Traditional Chinese Medicine, Xiamen Third Hospital, Fujian, Xiamen, China.; ^3^Department of Ultrasound Medicine, Zhuhai People's Hospital, The Affiliated Hospital of Beijing Institute of Technology, Zhuhai Clinical Medical College of Jinan University, China.; ^4^College of Integrated Chinese and Western Medicine, Hebei University of Chinese Medicine, Shijiazhuang, Hebei, China.; ^5^State Key Laboratory for Innovation and Transformation of Luobing Theory, Shijiazhuang, China.; ^6^New Drug Evaluation Center, Shijiazhuang Yiling Pharmaceutical Co., Ltd, Shijiazhuang, Hebei, China.

**Keywords:** Multimodal imaging, BZBS capsules, brain function, aging, brain degeneration

## Abstract

Aging reduces cerebral blood flow and impairs vascular function, yet how hemodynamics and brain function change during aging or after drug intervention remains unclear. Here we employed multimodal imaging, including macroscale MRI, mesoscale laser speckle contrast imaging, and microscale OCT angiography, to investigate cerebral hemodynamic and structural alterations in both naturally aged and D-galactose-induced aging mouse models. We also examined the effects of BZBS capsules on age-related vascular and cognitive decline. Aging caused a progressive loss of vascular density and decreased perfusion in primary and collateral vessels; BZBS treatment markedly preserved vascular integrity and restored perfusion. Moreover, BZBS administration enhanced memory function, increased superoxide dismutase (SOD) activity, improved antioxidant capacity, reduced malondialdehyde (MDA) levels, and alleviated white matter degeneration, collectively contributing to improved brain function and delayed aging. These findings identify BZBS capsules as a promising therapeutic candidate for mitigating brain aging and extending cognitive healthspan. Our study provides a strong foundation for future clinical trials to evaluate their translational potential in aging-related neurovascular dysfunction.

## INTRODUCTION

Aging causes physiological functions to decline and raises disease risk. As populations age globally, conditions such as cardiovascular disease [1], cancers [2], and neuro-degenerative disorders [3] pose increasing threats. The World Health Organization predicts that by 2050, 22 % of people will be 60 years or older—up from 12 % in 2015, totaling nearly 2 billion individuals. Although aging is irreversible, its progression can be slowed. Interventions on diet, mitochondrial health [4], protein homeostasis [5], and gut microbiota [6] show promise in delaying aging phenotypes. Compounds such as rapamycin, senolytics, metformin, acarbose, spermidine, NAD^+^ enhancers, and lithium exhibit anti-aging effects [8], and various herb-derived small molecules also mitigate neurodegenerative changes [7-11]. These findings underscore the potential of targeting specific pathways to improve health outcomes and reduce the burden of aging-associated diseases.

Brain aging is a critical aspect of the aging process, profoundly affecting cognitive function, emotional well-being, and independence. It is also closely linked to various neurodegenerative diseases [12]. The molecular mechanisms underlying brain aging are complex and multifaceted, highlighting the need for therapeutic strategies that target multiple pathways. Traditional Chinese Medicine (TCM), primarily derived from natural plant extracts, has gained attention for its role in treating neurodegenerative diseases [7, 13]. The BaZiBuShen Capsule (BZBS), composed of 16 herbal ingredients [14], has shown clinical efficacy in alleviating fatigue, treating impotence and male infertility, tonifying kidney function, and delaying aging [14, 15]. Huang et al. [16] conducted a chemical analysis of the BZBS capsule and identified compounds including chlorogenic acids, flavonoids, phenylethanoid glycosides, coumarins, lignans, and the terpenoid catalpol. These compounds contain various natural plant-based ingredients with anti-aging properties and are able to prevent age-related cognitive impairment. Moreover, recent studies indicate that BZBS not only slows cognitive decline by inhibiting microglial activation and cellular senescence [11] but also supports redox homeostasis, reduces immune senescence, and preserves telomerase activity and telomere length [17]. However, limited research has explored the effects of BZBS on cerebral hemodynamics, vascular remodeling, brain parenchymal changes, and anti-aging biomarkers. D-galactose, a widely used chemical reagent, has been employed to study brain aging mechanisms and evaluate anti-aging therapies by constructing rapid aging models in mice. Prolonged administration of high doses of D-galactose induces physiological and biochemical changes that closely resemble natural aging, including oxidative stress, inflammation, and neurodegeneration. This model offers a reliable and efficient approach to simulate the aging process within a shorter timeframe, making it an invaluable tool for exploring the mechanisms of aging and developing anti-aging interventions [17, 18].

Current methods for investigating the molecular interactions and therapeutic effects of BZBS capsules primarily include immunohistochemistry, biomarker assays, serum analysis, and fluorescence microscopy [11, 17]. These techniques monitor the distribution and accumulation of active compounds in the body, providing insights into how the capsules influence targeted tissues, regulate immune responses, and support organ function. However, in vivo multiscale imaging studies on the long-term effects of BZBS capsules in mice remain insufficient. Specifically, there is limited knowledge regarding the sustained use of BZBS and its impact on cerebral hemodynamics, vascular networks, antioxidant capacity, memory, and overall brain function. Understanding these long-term effects is essential for evaluating the therapeutic potential of BZBS capsules and elucidating their mechanisms of action in the context of age-related health and disease management.

**Figure 1.**
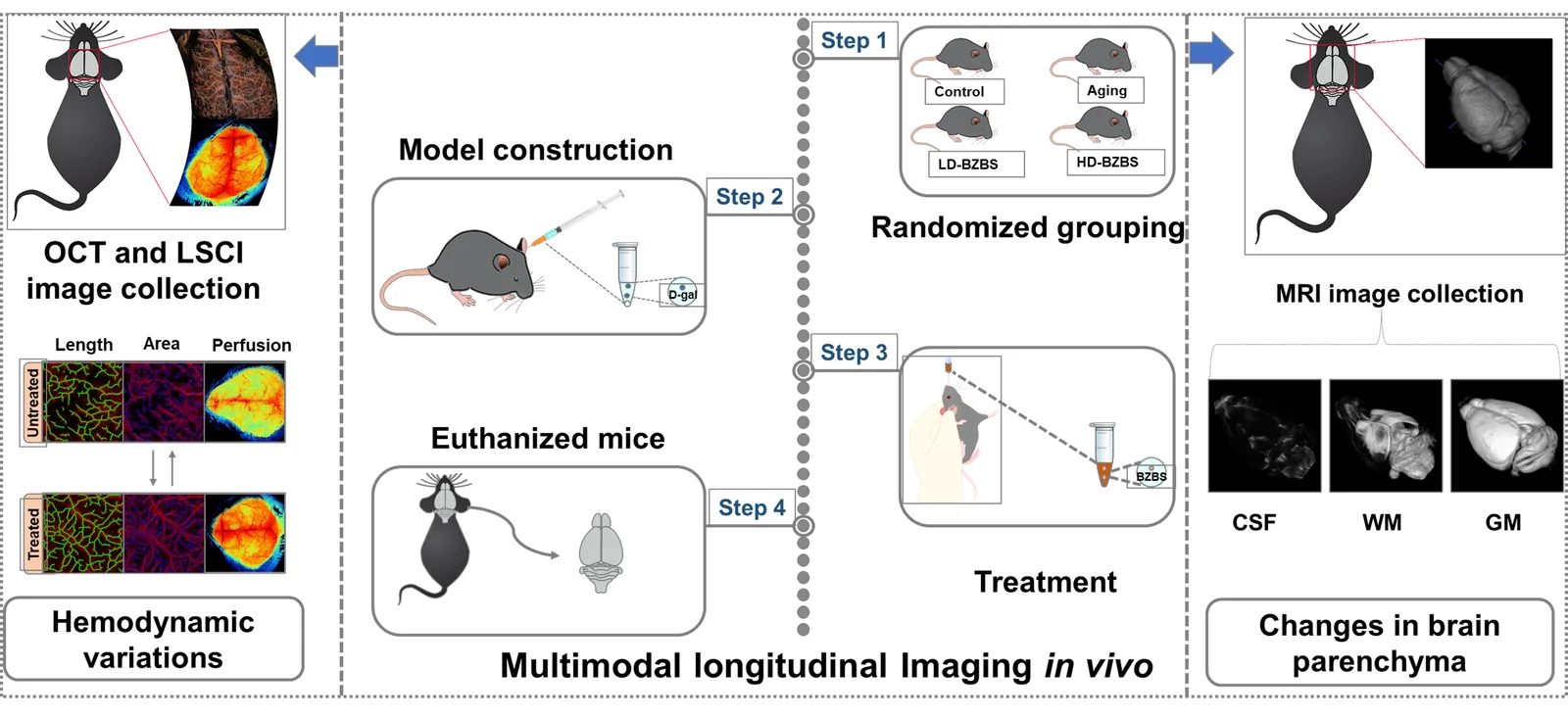
Overview of the experimental design, including aging, treatment with LD-BZ and HD-BZ, and assessment of vascular area, vessel length, and blood perfusion in the brain.

In this study, we propose a multimodal imaging paradigm to evaluate the effects of BZBS on cerebral hemodynamics, vascular remodeling, brain parenchyma, and brain-related behavioral outcomes and biomarkers in vivo using a D-galactose-induced aging mouse model. The results showed that cerebral blood perfusion in the aging group was significantly lower than in the control, low-dose treatment, and high-dose treatment groups (*P* < 0.05). Additionally, the white matter volume in the high-dose treatment group was significantly greater than in the aging group (*P* < 0.05) and closely resembled levels observed in the control group. Biochemical analyses further revealed that BZBS treatment significantly enhanced antioxidant capacity in aging mice. Moreover, H&E staining demonstrated that the hippocampus in the aging group exhibited the highest percentage of deeply stained areas (*P* < 0.05). Collectively, these findings indicate that BZBS delays brain aging by improving cerebral blood circulation, modifying brain parenchymal structures, and enhancing antioxidant defenses. This study provides robust imaging evidence and insights into the potential mechanisms by which BZBS combat brain aging.

## MATERIALS AND METHODS

### Materials

The BaZiBuShen (BZBS) capsule was manufactured by Yiling Pharmaceutical Co., Ltd., Shijiazhuang, China (batch number: 20230201, production date: February 1, 2023). Carboxymethyl cellulose sodium was obtained from Sigma-Aldrich. D-galactose was obtained from Guangzhou Yunshan Biotechnology Co., Ltd. The assay kits for GPX, SOD, and MDA were provided by Nanjing Jiancheng Bioengineering Institute. The H&E staining reagents were supplied by Xiamen Minyan Biotechnology Co., Ltd.

### Experimental procedure

Drug preparation: BZBS was suspended in 0.5% carboxymethyl cellulose sodium (CMC-Na) and D-galactose was dissolved in sterilized distilled water (SDW). Mice received daily subcutaneous D-galactose injections (120 mg/kg) and oral gavage of BZBS.

### Animal models

Male C57BL/6J mice (3 months old) were obtained from the Xiamen University Laboratory Animal Center (Ethics Approval No. XMULAC20230308). Male C57BL/6J mice (3 months old) were acclimated for 30 days under standard conditions (20-26 °C, 40-70% humidity, 12 h light/dark; food and water ad libitum) and then randomized (n = 6 per group) into control, aging, LD-BZ, and HD-BZ groups. Over the next 8 weeks, the aging group and both LD and HD treatment groups received daily subcutaneous D-galactose (120 mg/kg), while controls received an equal volume of sterile water.

Intragastric dosing began in week 4 of modeling and continued for eight weeks: the LD group received 1 g/kg/day and the HD group 2 g/kg/day, while control and aging mice received an equivalent volume of 0.5% sodium carboxymethylcellulose. Baseline OCT, LSCI, MRI, behavioral tests, and serum samples were collected before modeling; OCT, LSCI, and MRI were repeated at weeks 4, 8, and study end, with behavioral assessments and serum sampling at the end, after which mice were euthanized and brains harvested for analysis (Fig. 2).

**Figure 2.**
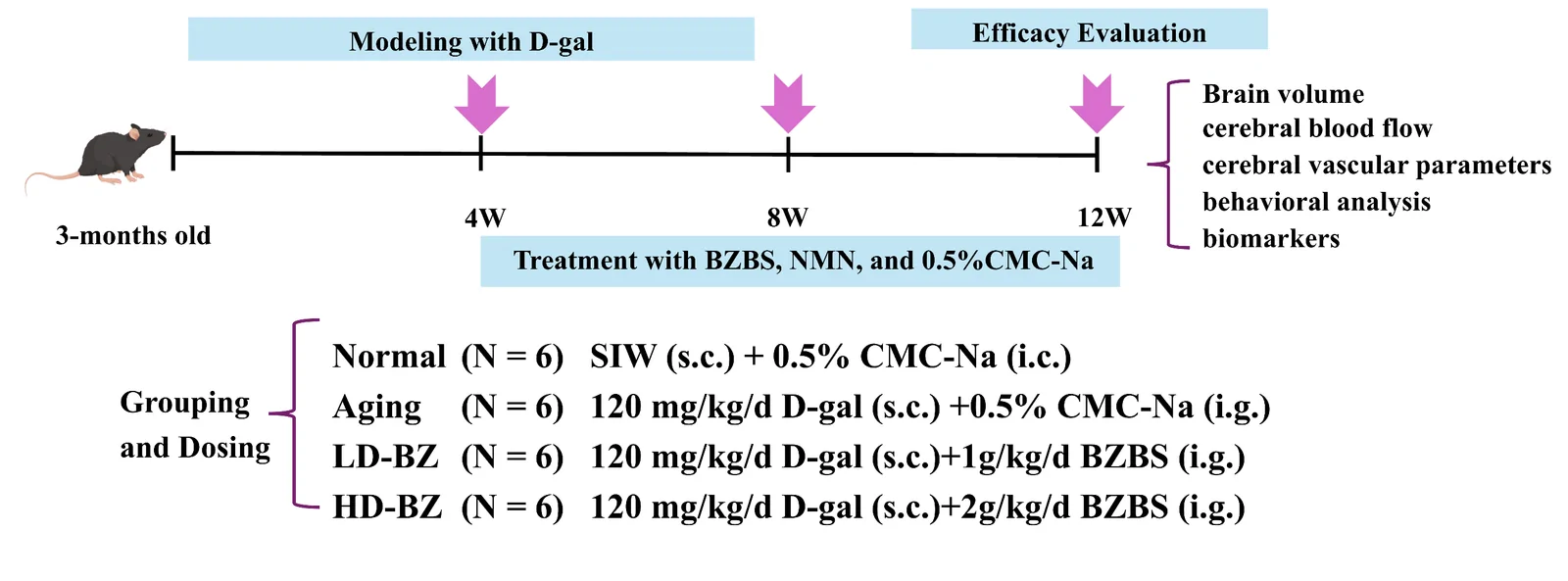
Experimental subgroups and their methodology for each group and the entire experimental timeline.

### In vivo optical coherence tomography angiography (OCTA)

The system is designed for high-resolution imaging of biological tissues. A custom high-resolution OCTA system (center wavelength 1060 nm; 200 kHz sweep rate; 100 nm bandwidth) provided a 10×10 mm field of view with 800 A-lines/B-scan and 800 B-scans/C-scan (20 repeats per region). For further details, our previous work [19, 20] provides a comprehensive description of the system. Mice were fixed on a custom-designed fixation platform and continuously inhaled a mixture of 2% isoflurane and 98% air throughout the experiment to maintain anesthesia. The scalp over the skull was trimmed with surgical scissors to facilitate OCT imaging. OCT scans were performed in weeks 0, 4, 8, and 12 for each mouse. Repeated OCT B-scan were processed using the split-spectrum amplitude-decorrelation angiography (SSADA) algorithm to obtain blood flow images, with vessels color-coded according to depth. OCTA images were processed using custom algorithms implemented in MATLAB (MathWorks, USA) and ImageJ, with the detailed workflow illustrated in Fig. 3. Vessel segmentation in OCTA images enables detailed quantification of vascular parameter changes [21]. Accordingly, we applied conventional image-processing algorithms to extract and quantify vessel area and length metrics from the OCTA images (Fig. 5 A). Briefly, two regions of interest were selected for each mouse. The images were processed sequentially using a Frangi vesselness filter (FrangiFilter2D), threshold segmentation, Canny edge detection, and morphological skeletonization to quantify vascular area and extract vessel skeletons. All steps were implemented in custom scripts written in MATLAB R2021a (MathWorks, Natick, MA, USA).

**Figure 3.**
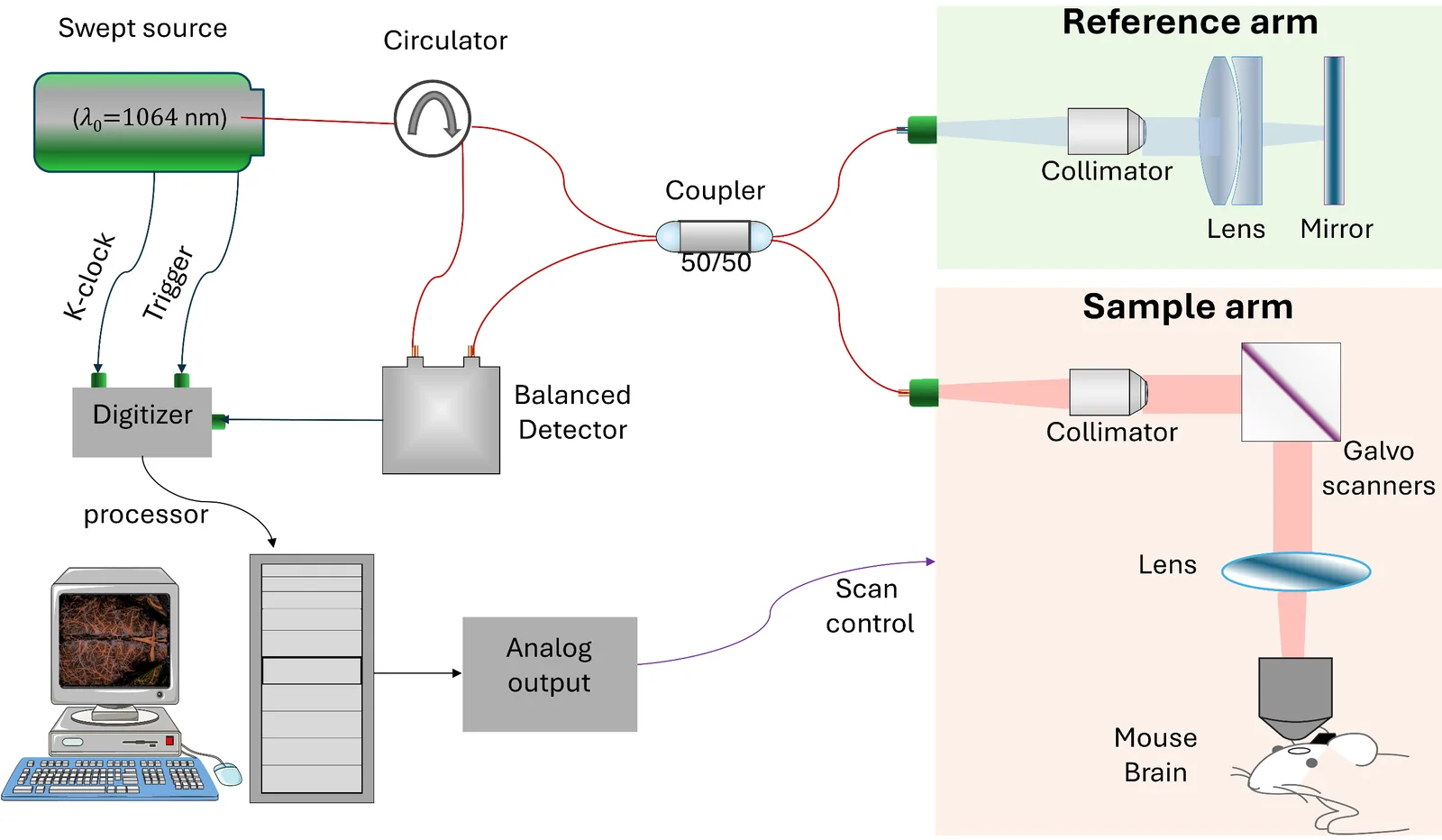
Schematic diagram of the optical coherence tomography (OCT) system.

### In vivo LSCI

Laser speckle contrast imaging was performed with a custom 2 cm × 2 cm, 1400 px × 1400 px system at 60 fps. Under 2% isoflurane anesthesia on a fixed platform, three ROIs (main, left, and right branch vessels) were defined; once perfusion stabilized, blood flow in each ROI was recorded for 20 s, and the mean value was taken as the final perfusion measurement.

### In vivo MRI

Multi-slice multi-echo sequence brain imaging was performed using a 9.4T MR scanner (Biospec, Bruker) with the following parameters: slices: 35; slice thickness: 0.5 mm; field of view: 2 cm × 2 cm; resolution: 256 px × 256 px. Mice were secured on a dedicated MRI platform and maintained under 2% isoflurane anesthesia throughout imaging. T2-weighted images were segmented by a trained U-Net model [22], registered to a mouse atlas [23], and processed in a modified FSL (version 6.0.6) [24] to segment gray matter, white matter, and cerebrospinal fluid. The volumes of gray matter, white matter, and CSF were calculated using the following formula:

(1) Volume=Voxels×Mean×V#1

where *Voxels* refers to the number of pixels of the corresponding tissue, *Mean* is the average partial volume, and *V* is the volume of a single voxel.

### Y-maze experiment

Mice were acclimated to the Y-maze room one day before testing. During a single 5-minute trial, each mouse was placed in the Y-shaped apparatus (45 cm × 10 cm × 15 cm) and allowed to explore freely while its movements were video-recorded; the maze was wiped with 75 % alcohol between trials. Behavioural parameters (time in each arm, speed, and arm transitions) were analysed using CleverSys TopScanLite (Clever Sys, Reston, VA, USA).

### SOD activity measurement

The SOD detection kit was provided by Nanjing Jiancheng Bioengineering Institute. Following the instructions, the measurement and control tube solutions were prepared, and after mixing in a constant temperature water bath for 40 minutes, a chromogenic agent was added. Distilled water was used for zeroing, and colorimetric analysis was conducted at a wavelength of 550 nm. The calculation of SOD activity is as follows:

(2) SOD activityUmL=(ODcontrol-ODtest)ODtest×50% #2

Where *_OD_control* is the absorbance value of the control group and *_OD_test* is the absorbance value of the test group.

### GSH-PX activity measurement

The GPX detection kit was provided by Nanjing Jiancheng Bioengineering Institute. Solutions were prepared according to the instructions. Distilled water was used for zeroing, and the absorbance was measured at a wavelength of 412 nm. The formula for calculating GSH-PX enzyme activity in serum is as follows:

(3) GSH-PX activityU=ODcontrol-ODtestODStandard-ODblank×c×d×t×c1#3

Where *_OD_control* is the absorbance value of the control group, *_OD_test* is the absorbance value of the test group, *_OD_standard* is the absorbance value of the standard sample, and *_OD_blank* is the absorbance value of the blank group. *c* is the concentration of the standard sample, *d* of the test group is the number of times of dilution, *t* is the reaction time, and *c*_1_ is the concentration of the protein of the sample to be tested.

### MDA concentration determination

The MDA detection kit was provided by Nanjing Jiancheng Bioengineering Institute. Solutions were prepared according to the requirements. Distilled water was used for zeroing, and the absorbance was measured at a wavelength of 532 nm. The formula for calculating MDA concentration in serum is as follows:

(4) MDAnmolmL=ODtest-ODcontrolODstandard-ODblank×c×d #4

Where *_OD_control* is the absorbance value of the control group, *_OD_test* is the absorbance value of the test group, *_OD_standard* is the absorbance value of the standard sample, and *_OD_blank* is the absorbance value of the blank group. *c* is the concentration of the standard sample, and *d* of the test group is the number of times of dilution

### Hematoxylin-eosin staining

At the end of the experiment, the mice were sacrificed, and cardiac perfusion was performed using 0.9% NaCl followed by 4% paraformaldehyde solution. The whole brain was then harvested and fixed in 4% paraformaldehyde, embedded in paraffin, and sectioned at 8 μm. Hematoxylin and eosin (H&E) staining was performed on all sections according to the manufacturer’s instructions (Servicebio). The stained sections were subsequently scanned using the Olympus VS200 automated slide scanning system (Olympus, Japan).

### Statistical analysis

Each experimental group included at least three biological replicates. Data are expressed as mean ± standard deviation (SD). Prior to statistical testing, the normality of each dataset was assessed by the Shapiro-Wilk test and homogeneity of variances by Levene’s test. Datasets meeting these assumptions were analyzed by one-way analysis of variance (ANOVA), followed by the Student-Newman-Keuls post hoc test for multiple comparisons. Statistical analyses were performed using SPSS version 27.0 (IBM Corp., Armonk, NY, USA). A p-value < 0.05 was considered statistically significant.

## RESULTS

### Brain volume changes

The most pronounced age-related change is reduced brain volume: progressive atrophy of gray and white matter with a compensatory increase in cerebrospinal fluid (CSF) volume. White matter (WM) volume loss is closely linked to declines in processing speed, executive function and memory. Studies of “superagers”—individuals whose WM microstructure is relatively preserved [25]—have demonstrated partial resistance to age-related cognitive decline. Structural analysis of mouse brains showed that D-galactose-induced aging significantly reduced white matter volume and increased cerebrospinal fluid volume, while total brain volume remained essentially unchanged (Fig. 4). BZBS treatment markedly mitigated WM volume loss—in the LD-BZ group, WM volume was virtually restored to control levels. Likewise, the rise in CSF volume was significantly diminished, with the HD-BZ group showing the greatest effect. No significant changes were observed in gray matter or whole-brain volume across any groups, suggesting that D-galactose-induced aging does not affect gray matter volume. Although the LD-BZ group did not differ significantly from the HD-BZ groups, its intermediate volumetric values may reflect suboptimal drug saturation at this dose. Together, these findings indicate that BZBS effectively alleviates D-galactose-induced WM loss in a dose-dependent manner.

**Figure 4.**
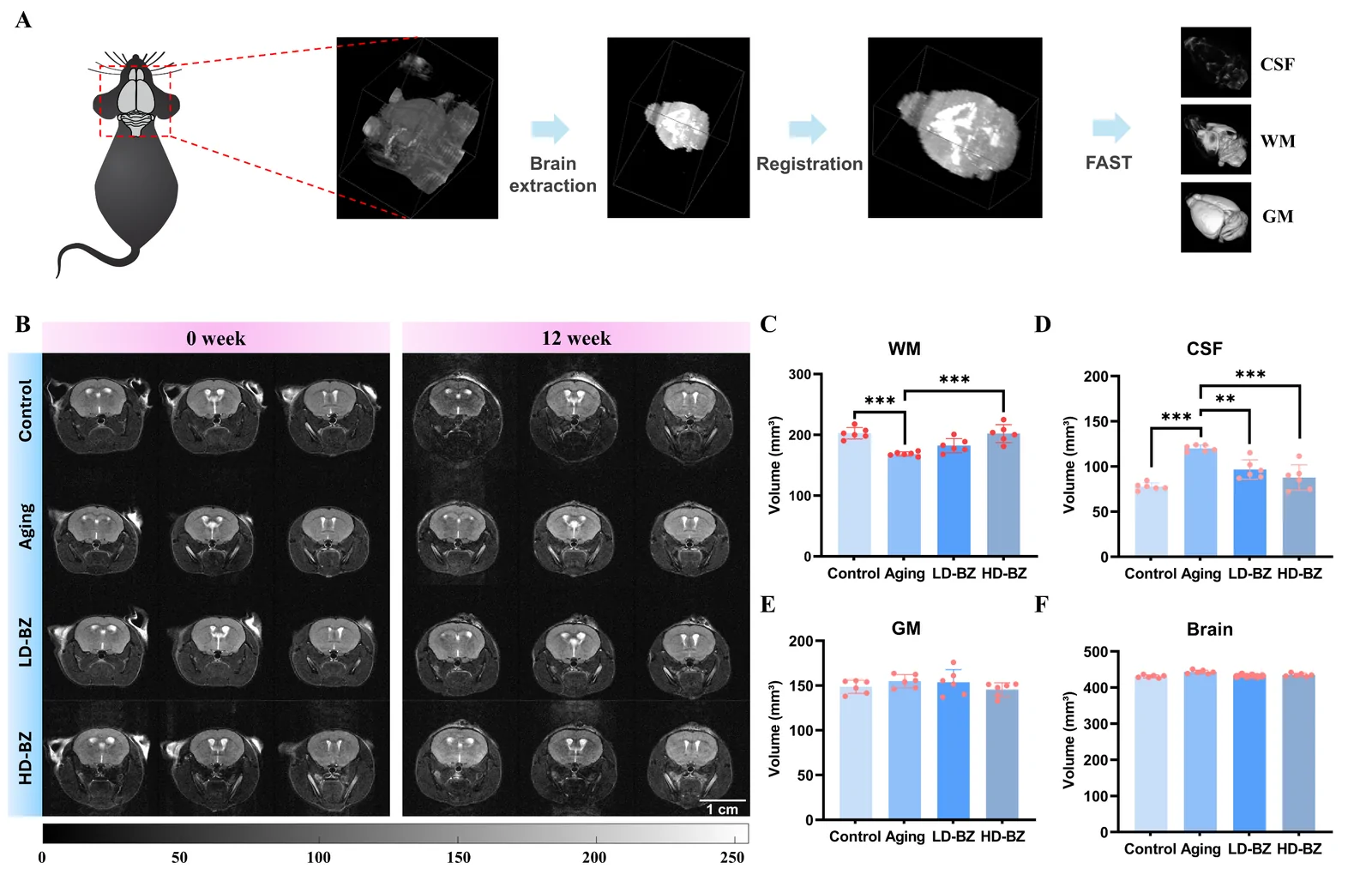
**Age-related changes in brain parenchyma and the effects of BZBS on brain parenchyma**. (**A**) MRI image processing workflow; (B) MRI images of the corpus callosum at the start of the experiment and the end of treatment for the Control, Aging, LD-BZ, and HD-BZ groups; (C, D, E, and F) The volume comparisons of white matter, cerebrospinal fluid, gray matter, and total brain at the end of treatment, respectively (N = 6 per group). Group differences were assessed by one-way ANOVA with Student-Newman-Keuls post-hoc test (* P < 0.05, ** P < 0.01, *** P < 0.001).

### Cerebral vascular changes

As aging advances, cerebral microvasculature undergoes marked remodelling—characterized by reduced vessel density and length, vessel-wall thickening, and luminal narrowing [26], which together impair cerebral perfusion and oxygen delivery. These structural alterations lead to capillary rarefaction and exacerbate vascular dysfunction, ultimately impairing neuronal activity and homeostatic regulation. In this study, we employed OCT to longitudinally image the cerebral vasculature in mice at defined time points, enabling quantitative assessment of dynamic structural remodelling. Figure 5 shows that microvessel density declined progressively with age across all mouse groups, with the aging group displaying the most pronounced reduction in vascular area. From week 8 onward, mice in the aging group exhibited significant decreases in both vascular area and vessel length (P < 0.05). At 12 weeks, the mean reduction in vascular length was 5.5 mm in the control group, 2.6 mm in the LD-BZ group, and 5.2 mm in the HD-BZ group—each significantly less than the 10.0 mm reduction observed in the aging group. Similarly, vascular density declined by 2.8 % in the control group, 0.1 % in the LD-BZ group and 1.3 % in the HD-BZ group compared with a 5.2 % reduction in the aging group. Compared with the control group, the aging group showed a decrease of 4.7 % (95 CI: [0.4 %, 9.0 %]) in brain vascular area and 9.0 (95 CI: [1.4, 16.7]) mm in vessel length at 12 weeks. In contrast, the LD-BZ group exhibited decreases of 0.2 % (95 CI: [-4.3 %, 4.7 %]) and 1.7 (95 CI: [-4.1, 11.9]) mm, while the HD-BZ group showed increases of 0.7 % (95 CI: [-4.6, 6.0]) and 1.5 (95 CI: [-7.6, 10.5]) mm, respectively (Fig. 5 A). Overall, although cerebral vascular density declines with age, BZBS administration significantly ameliorated this loss.

**Figure 5.**
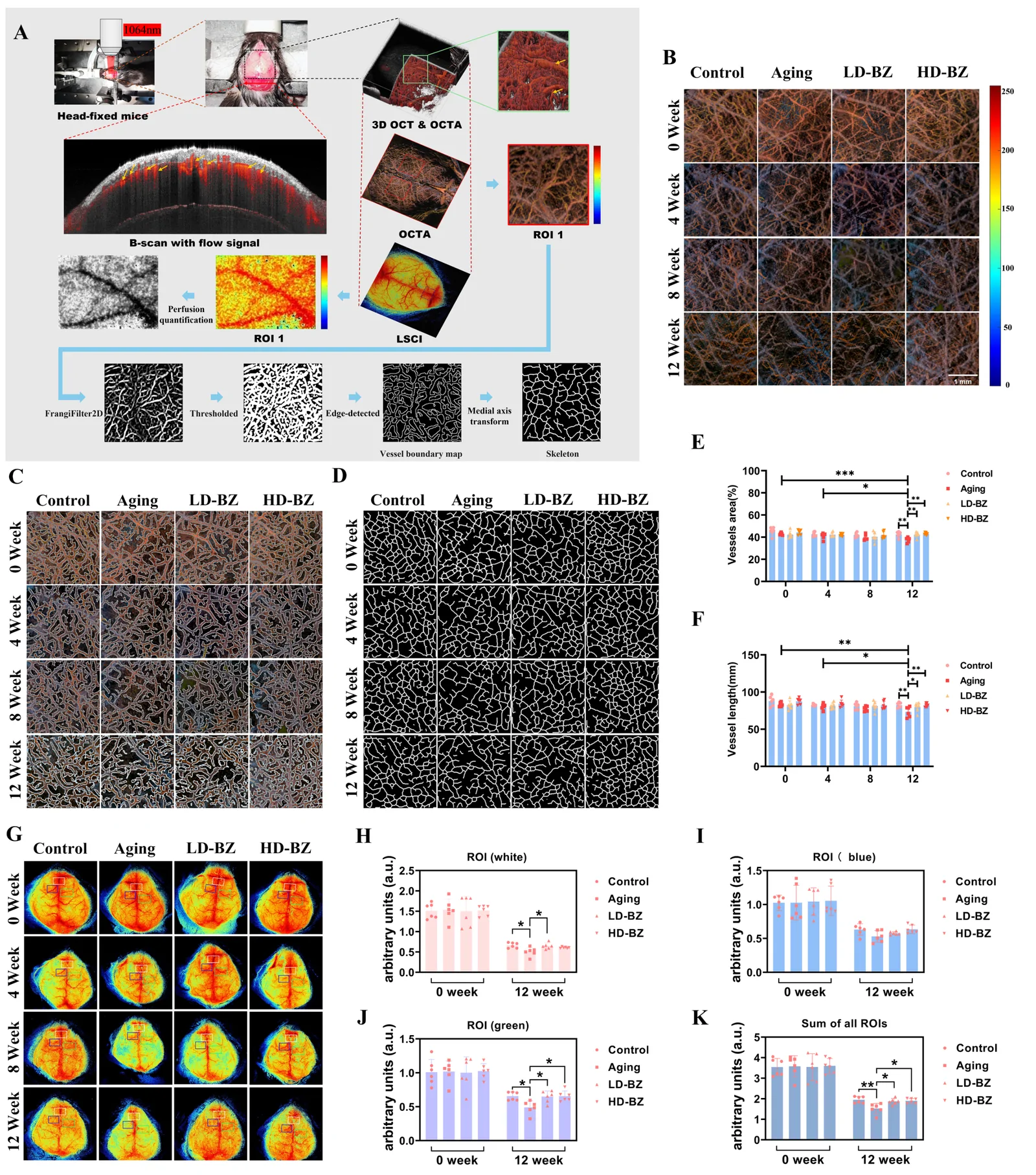
**Age-related changes in cerebral hemodynamics and the effects of BZBS on cerebral vasculature and blood flow**. (**A**) OCTA and LSCI processing workflow; (B) OCTA images of the brain at different time points for the control, aging, LD-BZ, and HD-BZ groups, with an ROI area of 2.5 mm × 2.5 mm; (C) Corresponding vascular framework images for panel B; (D) Corresponding vascular skeleton images for panel B; (E) Proportion of vascular area relative to the entire ROI, with a total of two different ROI areas per mouse, each measuring 2.5 mm × 2.5 mm; F, Total vascular length within the two ROI areas. G, LSCI images of the brain at different time points for the control, aging, LD-BZ, and HD-BZ groups, with an ROI area of 15 mm × 15 mm; H, Blood perfusion within the white solid line area in panel G; I, Blood perfusion within the blue solid line area in panel G; J, Blood perfusion within the green solid line area in panel G; K, Total blood perfusion across all ROI areas. Group differences were assessed by one-way ANOVA with Student-Newman-Keuls post-hoc test (N = 6 per group; * P < 0.05, ** P < 0.01, *** P < 0.001).

We employed laser speckle contrast imaging (LSCI) to monitor microcirculatory dynamics in the murine brain, and quantified blood perfusion in three regions of interest (ROIs): the main trunk vessel and its left and right branches. As shown in Fig. 5 G, cerebral blood perfusion volume in each group of mice declined progressively with age. As shown in Fig. 5 H-K, all groups exhibited a significant reduction in blood perfusion, most pronounced in the main branch vessels. Perfusion decreased by 0.84 a.u. in the control group, 1.03 a.u. in the aging group, 0.85 a.u. in the LD-BZ group and 0.91 a.u. in the HD-BZ group. At the end of treatment, blood perfusion volume in the main branch vessels of the aging group was significantly lower than in the control and LD-BZ groups (P < 0.05). In the right branch vessels, perfusion in the aging group was also significantly reduced compared with the control, LD-BZ, and HD-BZ groups (P < 0.05). Across all ROIs, cerebral blood perfusion decreased by 1.59 a.u. in the control group, 1.67 a.u. in the LD-BZ group and 1.70 a.u. in the HD-BZ group, compared with a 2.05 a.u. reduction in the aging group. At week 12, relative to the control group, the aging group exhibited a 0.44 (95 CI: [0.18, 0.71]) a.u. reduction in total ROI perfusion, a 0.08 (95 CI: [0.19, 0.34]) a.u. reduction in side-branch perfusion, and a total perfusion decrease of 0.04 (95 CI: [0.22, 0.31]) a.u. (P < 0.05). Both the LD-BZ and HD-BZ groups-maintained perfusion levels comparable to controls (Fig. 6B). Consequently, D-galactose-induced aging caused a progressive decline in cerebral blood perfusion, most pronounced in the major branch vessels. BZBS administration effectively mitigated this perfusion deficit.

**Figure 6.**
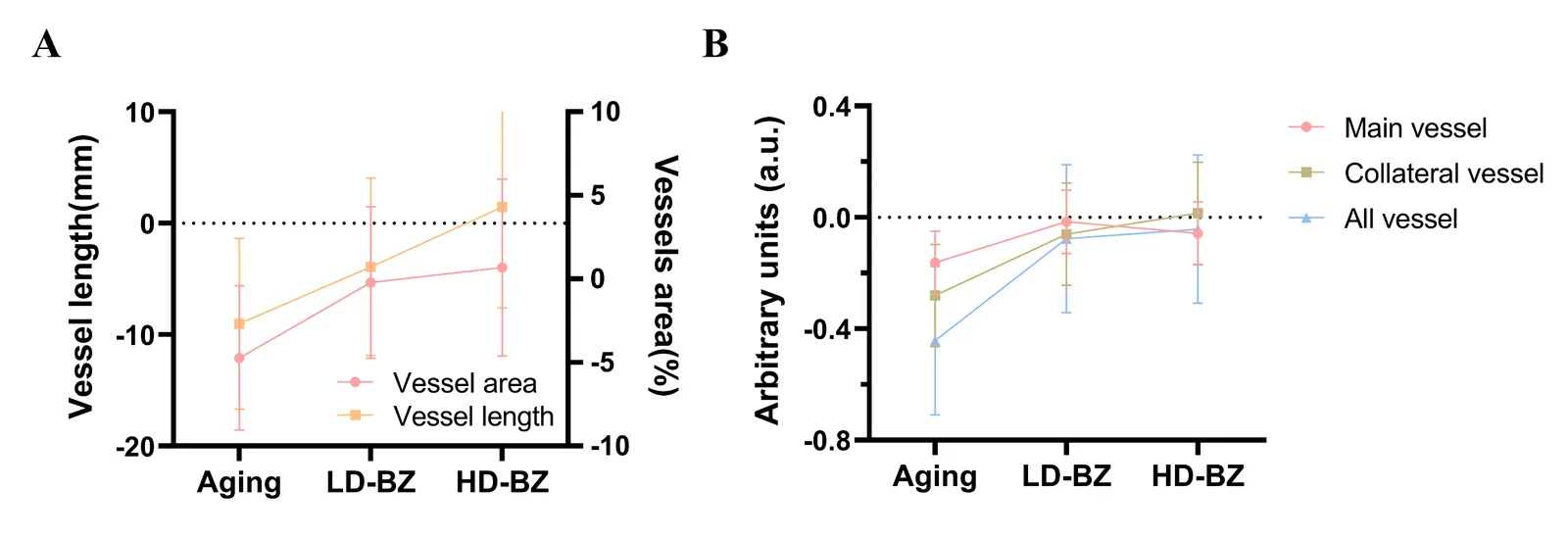
**Effects of BZBS on vasculature and blood flow**. Changes at week 12 in vascular area and vessel length (A) and in blood perfusion volume (B) in the aging, LD-BZ, and HD-BZ groups relative to the control group.

### Memory function test

To assess spatial working memory, we employed a Y-maze test at baseline (week 0) and at week 12. Mice were acclimated to the testing room 24 hours before each trial. During the 5-minute acquisition phase, one arm of the maze (“novel arm”) was blocked; mice were placed at the distal end of the start arm and allowed to explore the two open arms. After a 30-minute intertrial interval, mice were returned to the maze with all three arms open for a 5-minute test session. Trajectories were recorded and scored for spontaneous alternation—defined as consecutive entries into three different arms (e.g., ABC, BCA). Spontaneous alternation (%) was calculated as:

(5) Correct alternation%=Number of correct alternationsTotal number of alternations#5

As shown in Figure 7, at baseline there were no significant differences among groups in spontaneous alternation rate, mean locomotor speed, or total distance travelled. At week 12, the aging group exhibited significantly lower alternation rates compared with controls (P < 0.001), the low-dose group (P < 0.001), and the high-dose group (P < 0.01). Although the alternation rate of the low-dose group did not differ significantly from the aging group, it lay between the aging and high-dose groups; the high-dose group demonstrated a significantly higher alternation rate than the aging cohort. Moreover, mean locomotor speed and total distance traveled at week 12 were markedly reduced in the aging group relative to the control, LD-BZ, and HD-BZ groups (P < 0.05 for all comparisons), with the LD-BZ and HD-BZ groups showing slightly lower values than the control group, though these differences did not reach statistical significance. Thus, while BZBS treatment does not fully reverse D-galactose-induced memory deficits, high-dose BZBS significantly attenuates both cognitive decline and declines in locomotor activity.

**Figure 7.**
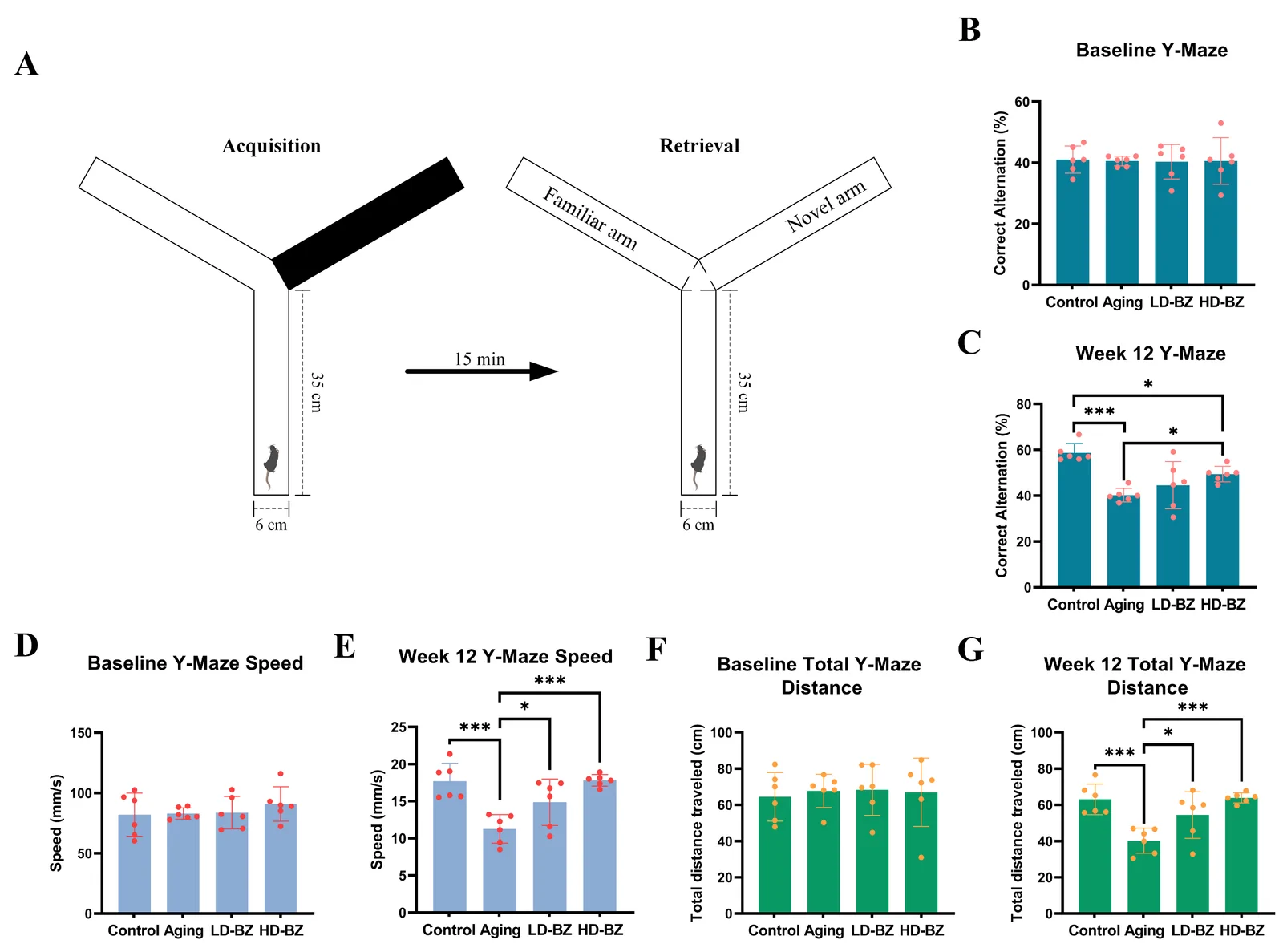
**Effects of BZBS on memory function in mice**. (**A**) Schematic diagram of the Y-maze; B, Correct alternation rate in each group at the initial stage; C, Correct alternation rate in each group at the end of treatment. Group differences were assessed by one-way ANOVA with Student-Newman-Keuls post-hoc test (N = 6 per group; * P < 0.05, ** P < 0.01, *** P < 0.001).

### Sections of the hippocampus

The hippocampus is among the most critical brain regions governing cognitive and memory functions [27, 28]. During aging, hippocampal neuronal density declines and nuclear staining becomes increasingly intense [29]. Notably, pyknotic neurons in the CA1 region correlate with impairments in synaptic plasticity and deficits in spatial learning and memory in rodent models of aging [30]. In hematoxylin-and-eosin-stained sections, these neurons display hallmark features of apoptotic or necrotic cell death—namely, hyperchromatic chromatin condensation, nuclear shrinkage (pyknosis), and intensified eosinophilia of the cytoplasm. Following the completion of behavioral experiments, we performed hematoxylin and eosin (H&E) staining on the hippocampal region of the mice to further investigate the reasons for the decline in memory function. As shown in Fig. 8, compared to the control group, the aging group exhibited more irregular cell shapes, disorganized cell arrangements, and darker nuclear coloration. These abnormalities were also present in both the low-dose and high-dose treatment groups, though to a lesser extent than in the aging group. Additionally, we quantitatively assessed the proportion of irregular cells in the CA1 region and dentate gyrus (DG) of the hippocampus using the following formula to calculate the proportion of densely stained cells:

$$\mathrm{Darker nucleoplasmic}\left(\%\right)=\frac{\mathrm{Darker nucleoplasmic area}}{\mathrm{CA}1\mathrm{area}+\mathrm{DG area}}$$

As mentioned previously, the proportion of densely stained cells in the aging group was significantly higher than that in the other three groups, with no statistically significant differences observed among the remaining groups. This suggests that BZBS can effectively delay D-galactose induced hippocampal damage and functional decline.

**Figure 8.**
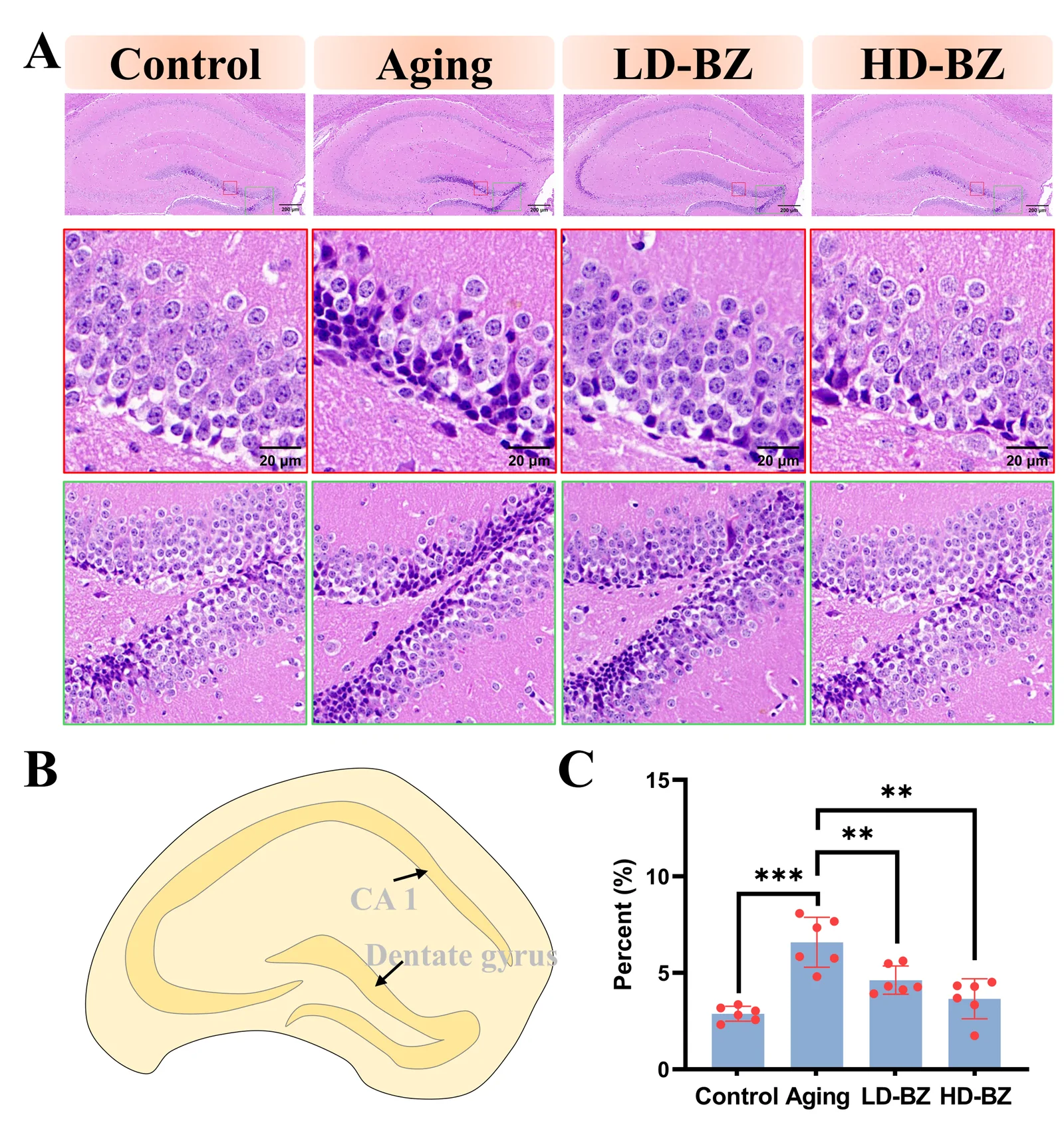
**Hippocampal H&E staining at treatment completion**. (**A**) H&E staining of the hippocampus in the Control, Aging, LD-BZ, and HD-BZ groups at the end of treatment, with the second row showing magnified images of the blue solid line area in the first row; (B) Schematic diagram of the mouse hippocampal structure; (C) Proportion of deeply stained cells in the CA1 region and dentate gyrus. Group differences were assessed by one-way ANOVA with Student-Newman-Keuls post-hoc test (N = 6 per group; * P < 0.05, ** P < 0.01, *** P < 0.001).

### Biochemical Indicators

Glutathione peroxidase (GPX), superoxide dismutase (SOD), and malondialdehyde (MDA) are key biomarkers of oxidative stress and aging [31-33]. GPX and SOD scavenge excess reactive oxygen species, protecting cells from oxidative damage, whereas MDA—an end product of lipid peroxidation—reflects the extent of such damage. We measured serum GPX, SOD, and MDA levels at baseline and after treatment (Fig. 9). Before intervention, no intergroup differences were observed (Fig. 9A-C). After 12 weeks, GPX activity remained unchanged across all groups, indicating minimal impact of D-galactose-induced aging on this enzyme. In contrast, SOD activity was significantly reduced in the aging group versus controls, whereas both low- and high-dose BZBS treatments restored SOD activity to levels comparable with controls (P < 0.01), with no significant difference between doses. MDA concentration was elevated in the aging group relative to all other groups, while low- and high-dose BZBS both prevented this rise, again without dose-dependent differences. These findings demonstrate that BZBS effectively counters age-related declines in antioxidant defences and lipid peroxidation, with low-dose treatment achieving maximal benefit.

**Figure 9.**
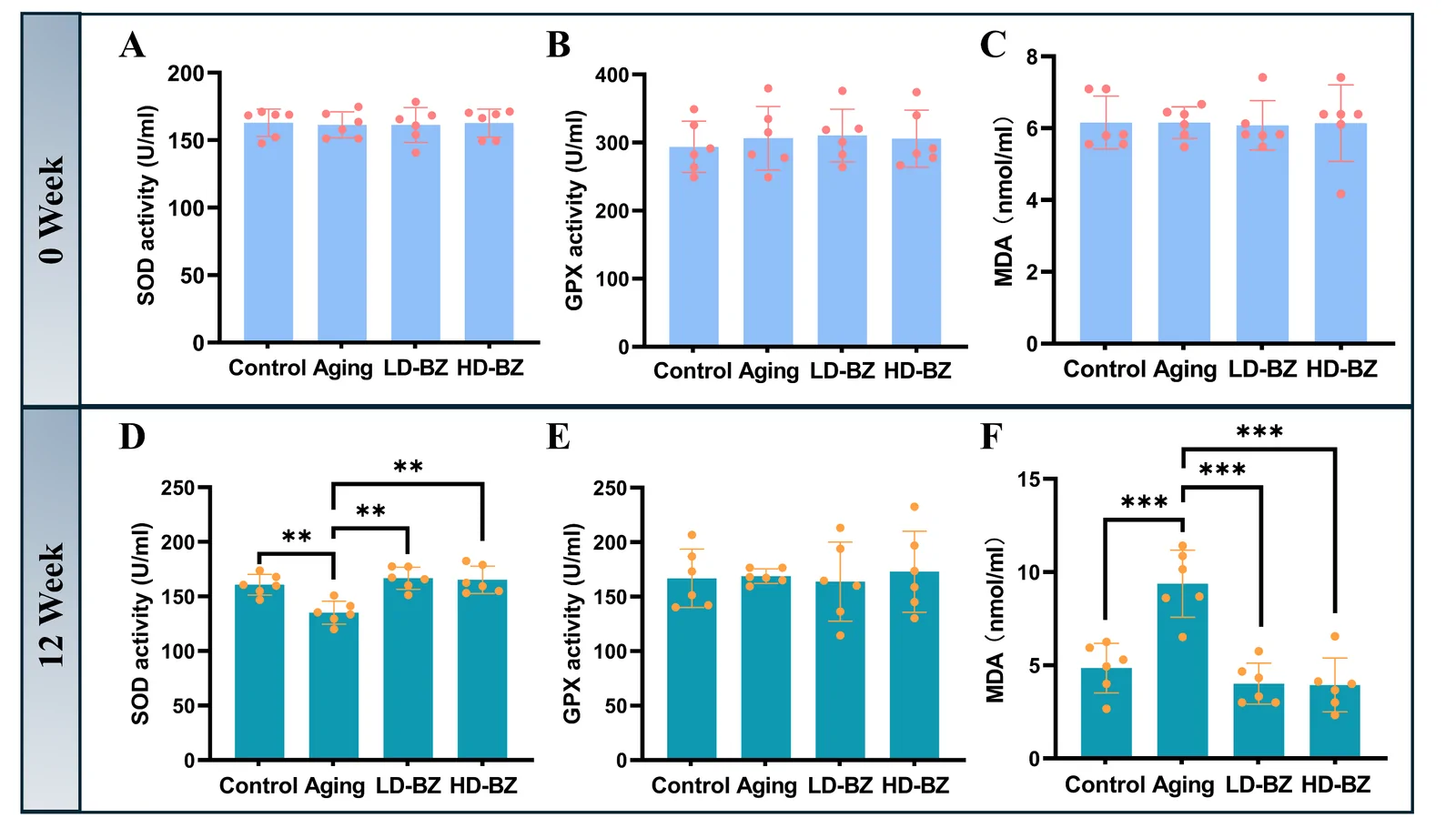
**Changes in serum oxidative markers (SOD, GPX, and MDA)**. (A, B, and C) The quantitative comparisons of serum SOD activity, MDA content, and GPX activity in each group of mice at the start of the experiment; (D, E, and F) The corresponding comparisons of serum SOD activity, MDA content, and GPX activity at the end of treatment. Group differences were assessed by one-way ANOVA with Student-Newman-Keuls post-hoc test (N = 6 per group; * P < 0.05, ** P < 0.01, *** P < 0.001).

## DISCUSSION

Brain aging critically contributes to neurodegenerative diseases through structural and parenchymal alterations, disrupted cerebral blood flow, reduced antioxidant defenses, and heightened inflammation. D-galactose is a commonly used chemical agent for constructing a rapid aging model, capable of inducing physiological and pathological changes that mimic natural aging, including elevated oxidative stress, inflammation, apoptosis, and cellular senescence. In this study, the anti-aging efficacy of BZBS capsules was evaluated by monitoring alterations in mouse brain parenchyma, local vascular parameters and cerebral blood flow perfusion, hippocampal morphology, and oxidative stress biomarkers. Results showed that BZBS capsules effectively alleviated d-galactose-induced reductions in cerebral blood flow, hippocampal dysfunction, decreased SOD activity, elevated MDA levels, white matter loss, and increased cerebrospinal fluid volume in mice, indicating that BZBS capsules can significantly mitigate aging-associated decline in brain function.

Consistent with previous studies, we found that D-galactose-induced aging led to a reduction in WM volume and an increase in CSF volume [34, 35]. The reduction in WM volume is associated with aging of the nervous system, although the underlying mechanisms remain unclear [36]. However, after treatment with BZBS, the decrease in WM volume and the increase in CSF volume were significantly attenuated. Moreover, the attenuation effect was dose-dependent, with the high-dose BZBS treatment showing better protection against parenchymal changes than the low-dose group, although neither exceeded the control group. Thus, BZBS capsules can macroscopically delay brain aging.

To further confirm the changes in brain parenchyma, we performed H&E staining of the hippocampal region. The results showed that, as in previous studies [37], D-galactose-induced aging mice exhibited irregular hippocampal cell arrangement and a higher proportion of deeply stained nuclei (hippocampal pathological changes). BZBS treatment also inhibited these hippocampal pathological changes, with the dose effect mirroring that observed for the inhibition of WM volume reduction. Additionally, WM reduction was closely associated with hippocampal pathological changes [38]. Therefore, the potential mechanism of BZBS may involve attenuating white matter volume loss. Since the hippocampus is primarily responsible for memory function [39, 40], we further assessed the memory changes in mice using the Y-maze test. The performance of mice in the Y-maze was consistent with the degree of hippocampal pathology and with previous findings [19], further validating the therapeutic efficacy of BZBS.

Although the mechanisms underlying age-related white matter volume loss remain incompletely understood, cerebral perfusion plays a pivotal role in this process [41]. Therefore, we observed changes in brain vascular parameters and blood perfusion in mice from each group using OCT and LSCI systems. As described, BZBS significantly ameliorated age-related reductions in vascular area, vessel length, and perfusion. However, the inhibition of vascular parameters did not show a dose-dependent relationship, with the high-dose treatment group even showing slightly lower effects than the low-dose treatment group. We think that BZBS may reach a saturation point at the low dose, and further increasing the dose does not provide additional therapeutic benefits. The mechanisms underlying the reduction in WM volume remain unclear and are likely the result of multiple factors, whose effects may accumulate. BZBS plays only a partial role in this process and cannot fully prevent the reduction in WM volume. Moreover, for inhibiting WM volume shrinkage, high-dose treatment appears to be more effective than low-dose treatment.

In addition, oxidative stress and elevated levels of inflammation are key influencing factors in the aging process. By measuring the concentrations of SOD, GPX, and MDA, we found that the SOD levels in both the low and high dose treatment groups of BZBS were significantly higher than those of the aging group, and were consistent with those of the control group.The MDA levels were significantly lower than those of the aging group, and slightly lower than those of the control group, as n previous studies on BZBS [17]. However, there was no significant difference in GPX levels between the groups, suggesting that D-galactose-induced senescence may not lead to a decrease in GPX levels, and BZBS treatment did not lead to an increase in GPX levels, further supporting the conclusion that BZBS could not reverse senescence. It suggests that BZBS can effectively alleviate the decrease in antioxidant capacity caused by D-galactose-induced senescence. In conclusion, our study provides strong preclinical evidence supporting the anti-aging effects of BZBS in a mouse model. It showed significant inhibitory effects on aging-induced pathological changes in cerebral perfusion, SOD activity and MDA levels.

In summary, our study was conducted in mice and provides a visualized evaluation of BZBS capsule therapy, yet inherent limitations remain in extrapolating these animal data to clinical settings. Moreover, any candidate drug must undergo extensive preclinical testing before entering clinical trials. We employed a D-galactose-induced accelerated aging model, which, although widely accepted, does not fully recapitulate natural human senescence. Accordingly, in the next phase of our research, we will prioritize visualizing and validating the anti-aging effects of BZBS in naturally aged animal models. Furthermore, this work broadens the evidence base for BZBS’s anti-aging efficacy, offers robust support for its clinical translation, and introduces a novel framework for longitudinal monitoring of aging dynamics using multimodal imaging techniques.

## Conclusion

Our study demonstrates that BZBS significantly improves physiological and neurological deficits in D-galactose-induced aging mice. It restores cerebral blood perfusion, preserves hippocampal function, maintains SOD activity, reduces MDA levels, and protects white matter integrity. Low doses are effective for antioxidant and vascular maintenance, while higher doses better address severe impairments. Although BZBS delays brain aging, it cannot reverse it, underscoring the importance of early intervention.

## Data Availability

The data supporting the findings of this study are available from the corresponding author upon reasonable request.

## References

[1] Katan M, Luft A.Global burden of stroke. Thieme Medical Publishers. 208-211. 10.1055/s-0038-1649503 29791947

[2] Calcinotto A, Kohli J, Zagato E, Pellegrini L, Demaria M, Alimonti A (2019). Cellular senescence: aging, cancer, and injury. Physiol Rev, 99:1047-1078. 10.1152/physrev.00020.2018 30648461

[3] Zeppenfeld DM, Simon M, Haswell JD, D’Abreo D, Murchison C, Quinn JF, et al. (2017). Association of perivascular localization of aquaporin-4 with cognition and Alzheimer disease in aging brains. JAMA Neurol, 74:91-99. 10.1001/jamaneurol.2016.4370 27893874

[4] Fivenson EM, Lautrup S, Sun N, Scheibye-Knudsen M, Stevnsner T, Nilsen H, et al. (2017). Mitophagy in neurodegeneration and aging. Neurochem Int, 109:202-209. 10.1016/j.neuint.2017.02.007 PMC5565781 28235551

[5] Zhang Q, Wu X, Chen P, Liu L, Xin N, Tian Y, et al. (2018). The mitochondrial unfolded protein response is mediated cell-non-autonomously by retromer-dependent Wnt signaling. Cell, 174:870-883. 10.1016/j.cell.2018.06.029 PMC6086732 30057120

[6] Boehme M, Guzzetta KE, Bastiaanssen TFS, Van De Wouw M, Moloney GM, Gual-Grau A, et al. (2021). Microbiota from young mice counteracts selective age-associated behavioral deficits. Nat Aging, 1:666-676. 10.1038/s43587-021-00093-9 37117767

[7] Tewari D, Stankiewicz AM, Mocan A, Sah AN, Tzvetkov NT, Huminiecki L, et al. (2018). Ethnopharmacological approaches for dementia therapy and significance of natural products and herbal drugs. Front Aging Neurosci, 10:3. 10.3389/fnagi.2018.00003 PMC5816049 29483867

[8] Kim E-C, Kim J-R (2019). Senotherapeutics: emerging strategy for healthy aging and age-related disease. BMB Rep, 52:47. 10.5483/BMBRep.2019.52.1.293 PMC6386227 30526770

[9] Cui X, Lin Q, Liang Y (2020). Plant-derived antioxidants protect the nervous system from aging by inhibiting oxidative stress. Front Aging Neurosci, 12:209. 10.3389/fnagi.2020.00209 PMC7372124 32760268

[10] Ahmad S, Khan A, Ali W, Jo MH, Park J, Ikram M, et al. (2021). Fisetin rescues the mice brains against D-galactose-induced oxidative stress, neuroinflammation and memory impairment. Front Pharmacol, 12:612078. 10.3389/fphar.2021.612078 PMC7947859 33716741

[11] Ji C, Wei C, Li M, Shen S, Zhang S, Hou Y, et al. (2022). Bazi Bushen capsule attenuates cognitive deficits by inhibiting microglia activation and cellular senescence. Pharm Biol, 60:2025-2039. 10.1080/13880209.2022.2131839 PMC9590440 36263579

[12] Hou Y, Dan X, Babbar M, Wei Y, Hasselbalch SG, Croteau DL, et al. (2019). Ageing as a risk factor for neurodegenerative disease. Nat Rev Neurol, 15:565-581. 10.1038/s41582-019-0244-7 31501588

[13] Long F, Yang H, Xu Y, Hao H, Li P (2015). A strategy for the identification of combinatorial bioactive compounds contributing to the holistic effect of herbal medicines. Sci Rep, 5:12361. 10.1038/srep12361 PMC4510521 26198093

[14] Huang D, Wang X, Zhu Y, Gong J, Liang J, Song Y, et al. (2021). Bazi bushen capsule alleviates post-menopausal atherosclerosis via gper1-dependent anti-inflammatory and anti-apoptotic effects. Front Pharmacol, 12:658998. 10.3389/fphar.2021.658998 PMC8267998 34248622

[15] Li L, Chen B, An T, Zhang H, Xia B, Li R, et al. (2021). BaZiBuShen alleviates altered testicular morphology and spermatogenesis and modulates Sirt6/P53 and Sirt6/NF-κB pathways in aging mice induced by D-galactose and NaNO2. J Ethnopharmacol, 271:113810. 10.1016/j.jep.2021.113810 33508368

[16] Huang D, Wang X, Zhu Y, Gong J, Liang J, Song Y, et al. (2021). Bazi Bushen Capsule Alleviates Post-Menopausal Atherosclerosis via GPER1-Dependent Anti-Inflammatory and Anti-Apoptotic Effects. Front Pharmacol, 12:658998. 10.3389/fphar.2021.658998 PMC8267998 34248622

[17] Li L, Zhang H, Chen B, Xia B, Zhu R, Liu Y, et al. (2022). BaZiBuShen alleviates cognitive deficits and regulates Sirt6/NRF2/HO-1 and Sirt6/P53-PGC-1α-TERT signaling pathways in aging mice. J Ethnopharmacol, 282:114653. 10.1016/j.jep.2021.114653 34547420

[18] Shwe T, Pratchayasakul W, Chattipakorn N, Chattipakorn SC (2018). Role of D-galactose-induced brain aging and its potential used for therapeutic interventions. Exp Gerontol, 101:13-36. 10.1016/j.exger.2017.10.029 29129736

[19] Chowdhury AA, Gawali NB, Bulani VD, Kothavade PS, Mestry SN, Deshpande PS, et al. (2018). In vitro antiglycating effect and in vivo neuroprotective activity of Trigonelline in d-galactose induced cognitive impairment. Pharmacol Rep, 70:372-377. 10.1016/j.pharep.2017.09.006 29477946

[20] Huang D, Wang G, Mao J, Liu C, Fan Z, Zhang Y, et al. (2021). Intravital whole-process monitoring thermo-chemotherapy via 2D silicon nanoplatform: a macro guidance and long-term microscopic precise imaging strategy. Adv Sci, 8:2101242. 10.1002/advs.202101242 PMC8373095 34166580

[21] Xing J, Jiang S, Xu J, Guo D, Zhang J, Li P, et al. (2025). FS-Net collaborative flattening transformation for high performance 3D-to-2D vessel segmentation in OCTA images. Neurocomputing:130113.

[22] Ronneberger O, Fischer P, Brox T. U-net: Convolutional networks for biomedical image segmentation. Springer. 234-241.

[23] Ma Y, Hof PR, Grant SC, Blackband SJ, Bennett R, Slatest L, et al. (2005). A three-dimensional digital atlas database of the adult C57BL/6J mouse brain by magnetic resonance microscopy. Neuroscience, 135:1203-1215. 10.1016/j.neuroscience.2005.07.014 16165303

[24] Jenkinson M, Beckmann CF, Behrens TEJ, Woolrich MW, Smith SM (2012). Fsl. Neuroimage, 62:782-790. 10.1016/j.neuroimage.2011.09.015 21979382

[25] Garo-Pascual M, Zhang L, Valentí-Soler M, Strange BA (2024). Superagers Resist Typical Age-Related White Matter Structural Changes. J Neurosci, 44. 10.1523/JNEUROSCI.2059-23.2024 PMC11209667 38684365

[26] Bennett HC, Zhang Q, Wu Y-t, Manjila SB, Chon U, Shin D, et al. (2024). Aging drives cerebrovascular network remodeling and functional changes in the mouse brain. Nat Commun, 15:6398. 10.1038/s41467-024-50559-8 PMC11289283 39080289

[27] Moscovitch M, Nadel L, Winocur G, Gilboa A, Rosenbaum RS (2006). The cognitive neuroscience of remote episodic, semantic and spatial memory. Curr Opin Neurobiol, 16:179-190. 10.1016/j.conb.2006.03.013 16564688

[28] O'Keefe J, Dostrovsky J (1971). The hippocampus as a spatial map: preliminary evidence from unit activity in the freely-moving rat. Brain Res. 10.1016/0006-8993(71)90358-1 5124915

[29] Hu S, Li C-sR (2020). Age-related structural and functional changes of the hippocampus and the relationship with inhibitory control. Brain Sci, 10:1013. 10.3390/brainsci10121013 PMC7766783 33352718

[30] Rashad S, Saigusa D, Yamazaki T, Matsumoto Y, Tomioka Y, Saito R, et al. (2020). Metabolic basis of neuronal vulnerability to ischemia; an in vivo untargeted metabolomics approach. Sci Rep, 10:6507. 10.1038/s41598-020-63483-w PMC7162929 32300196

[31] Zhao F, Guo L, Wang X, Zhang Y (2021). Correlation of oxidative stress-related biomarkers with postmenopausal osteoporosis: a systematic review and meta-analysis. Arch Osteoporos, 16:1-10. 10.1007/s11657-020-00854-w 33400044

[32] Górny M, Bilska-Wilkosz A, Iciek M, Hereta M, Kamińska K, Kamińska A, et al. (2020). Alterations in the antioxidant enzyme activities in the neurodevelopmental rat model of schizophrenia induced by glutathione deficiency during early postnatal life. Antioxidants, 9:538. 10.3390/antiox9060538 PMC7346228 32575563

[33] Fallah F, Colagar AH, Saleh HA, Ranjbar M (2023). Variation of the genes encoding antioxidant enzymes SOD2 (rs4880), GPX1 (rs1050450), and CAT (rs1001179) and susceptibility to male infertility: a genetic association study and in silico analysis. Environ Sci Pollut Res, 30:86412-86424. 10.1007/s11356-023-28474-0 37405601

[34] Bontempi P, Podda R, Daducci A, Sonato N, Fattoretti P, Fiorini S, et al. (2021). MRI characterization of rat brain aging at structural and functional level: Clues for translational applications. Exp Gerontol, 152:111432. 10.1016/j.exger.2021.111432 34062262

[35] Ge Y, Grossman RI, Babb JS, Rabin ML, Mannon LJ, Kolson DL (2002). Age-related total gray matter and white matter changes in normal adult brain. Part I: volumetric MR imaging analysis. Am J Neuroradiol, 23:1327-1333. PMC7976241 12223373

[36] Kaya T, Mattugini N, Liu L, Ji H, Cantuti-Castelvetri L, Wu J, et al. (2022). CD8+ T cells induce interferon-responsive oligodendrocytes and microglia in white matter aging. Nat Neurosci, 25:1446-1457. 10.1038/s41593-022-01183-6 PMC9630119 36280798

[37] Zhang Q, Li X, Cui X, Zuo P (2005). D-galactose injured neurogenesis in the hippocampus of adult mice. Neurol Res, 27:552-556. 10.1179/016164105X25126 15978184

[38] Fiford CM, Manning EN, Bartlett JW, Cash DM, Malone IB, Ridgway GR, et al. (2017). White matter hyperintensities are associated with disproportionate progressive hippocampal atrophy. Hippocampus, 27:249-262. 10.1002/hipo.22690 PMC5324634 27933676

[39] Wilson IA, Ikonen S, Gallagher M, Eichenbaum H, Tanila H (2005). Age-associated alterations of hippocampal place cells are subregion specific. J Neurosci, 25:6877-6886. 10.1523/JNEUROSCI.1744-05.2005 PMC6725350 16033897

[40] Lisman J, Buzsáki G, Eichenbaum H, Nadel L, Ranganath C, Redish AD (2017). Viewpoints: how the hippocampus contributes to memory, navigation and cognition. Nat Neurosci, 20:1434-1447. 10.1038/nn.4661 PMC5943637 29073641

[41] Li Y, Choi WJ, Wei W, Song S, Zhang Q, Liu J, et al. (2018). Aging-associated changes in cerebral vasculature and blood flow as determined by quantitative optical coherence tomography angiography. Neurobiol Aging, 70:148-159. 10.1016/j.neurobiolaging.2018.06.017 PMC6119107 30007164

